# Physiological Indicators and Production Performance of Dairy Cows With Tongue Rolling Stereotyped Behavior

**DOI:** 10.3389/fvets.2022.840726

**Published:** 2022-02-25

**Authors:** Fuyu Sun, Qingyao Zhao, Xiaoyang Chen, Guangyong Zhao, Xianhong Gu

**Affiliations:** ^1^State Key Laboratory of Animal Nutrition, Institute of Animal Science, Chinese Academy of Agricultural Sciences, Beijing, China; ^2^College of Animal Science and Technology, China Agricultural University, Beijing, China

**Keywords:** welfare, stereotypy behavior, lactation, stress state, metabolism

## Abstract

Non-nutritive oral behaviors, especially tongue rolling, are prevalent in the stabled cow population. These behaviors mean that the environment or management process might not suit the cows, suggesting low welfare. However, few researches have reported the physiological indicators or production performance of dairy cows with the stereotyped behavior. This study aimed to determine physical conditions, daily activity, rumen fermentation, and milk production of cows with tongue-rolling behavior. Three hundred and fifty nine Holstein cows in the same barn and lactation stage were subjected to scan sampling behavior observations 126 times for 7 days. Ten cows with high-frequency tongue-rolling behavior (TON) and 10 cows without abnormal oral behavior (CON) were selected for further study. Serum sample, ruminal fluid, milk sample, and behavior record video of TON and CON cows were collected. TON cows had more drinking behavior and more stable lying behavior than the CON cows during the daytime. The body condition score of the TON cows decreased, while the milk yield, yield of milk fat, protein, and lactose in the study period increased. The TON cows had lower ruminal fluid pH, acetate/propionate ratio, and total volatile acid. The bacterial diversity in the ruminal fluid was not different between the two groups. Compared to CON cows, the TON cows had a higher level of serum stress indicators, such as cortisol, thyroid hormone, and norepinephrine, which positively correlated to the frequency of tongue-rolling behavior. Meanwhile, the TON cows had a higher level of lactate dehydrogenase, serum glucose, total triglyceride, total cholesterol, and Interleukin 6. Overall, it means they suffer from higher levels of stress and have higher energy metabolism for a long time when cows show tongue-rolling behavior. TON cows had suffered a higher stress level and had higher energy metabolic status for a long time. The TON cows might have better heat tolerance to the thermal environment by more lying and drinking time. Our data revealed the changes in milk production, physiological stress indicators of dairy cows with high-frequency tongue rolling behavior, which will provide essential knowledge for the in-depth understanding of tongue rolling behavior in dairy cows.

## Introduction

Stereotyped behavior, defined as repetitive, rhythmic behavioral activities with no apparent functional characteristics, is usually considered a substitute activity expressed when natural behavior cannot be expressed or animals are subjected to stressors. For cows, if stereotyped behavior occupied more than 10% of the time of waking life or it caused bodily injury, then this behavioral state can be said to be detrimental to the welfare of the animal (1). Compared to other species, a ruminant typically spends more than one-half of their day with its mouth (oral cavity) and tongue in motion, grazing or regurgitating (cud chewing) (2). At all stages of the cow growth, the most common behavioral pattern that is repetitive and fixed and fits the criteria of stereotypies is tongue-rolling by far (3), which consists of a repeated turning and extending of the tongue outside or sometimes inside the open mouth (4). Generally, researchers use the occurrence of stereotypic behaviors to identify animals as being housed in an unsuitable environment or feeding management (1) or suffering from welfare problems such as mental stress (5). The poorer the captive environment (poor abundance) and the smaller the space, the more likely stereotypic behaviors are to occur in animals. Although stereotypic behavior does not necessarily occur in stressed cows, it reflects the fact that cows may be in a prolonged state of physical and mental stress (6).

Lindström and Redbo (7) found that cows with low rumen content and short duration of eating spent longer time on rolling tongue. By comparing the occurrence and development of stereotyped oral behaviors, recent studies delineated that specific roughage dietary components (barley straw, wheat straw, and maize silage) or high roughage ratios may reduce non-nutrition oral behavior performance in cows (8–10). Based on this aspect, oral stereotypic behavior could be considered as a possible alternative to ruminant idle activity caused by inadequate roughage intake. But whether the rumen fermentation of dairy cows with tongue-rolling behavior was consistent with a series of changes resulting from insufficient roughage feeding is lacking research data. Redbo (3) found that the blood baseline cortisol and adrenocorticotropic hormone in dairy calves housed in tether stalls was related to individual stereotypy levels, and tethered animals that exhibit high frequency of stereotypic behavior had high heat production and metabolic rates (11). Researchers have also reported the etiologies and influencing factors of the expression of stereotypic behavior in other animals such as mice (12), pigs (13), horses (14, 15), and rhesus monkeys (16). The relationship between various types of stereotypic behaviors and various triggering factors reported in studies lacks consistency, mainly due to the multiple sources of stereotypic behavior development, the different ways in which the body perceives the environment, and the complex control of the neuroendocrine system. Thus, the occurrence of tongue rolling behavior in cows may result from multiple factors acting together (5). To supplement relevant data information, sampling and analyzing the target stereotyped behavioral cows from multiple perspectives such as nutritional metabolism, environmental heat tolerance, production performance, and blood physiology is necessary.

This was an observational study, without any hypothesis. In this study, we selected typical cows with rolling tongue behavior and cows without abnormal behavior as the sampling target by observing hundreds of dairy cows with scan sampling observations (recording the activity of cows at pre-selected time intervals) (17). Then we compared the differences in performance, ruminal fermentation, and serum stress physiology indicators between cows with tongue-rolling behavior and cows without abnormal behaviors, aimed to add essential information to the understanding of the tongue-rolling behavior in dairy cows. Establishing correlations between tongue rolling behavior and production performance or physiological indicators could indicate information for dairy cattle state diagnosis.

## Materials and Methods

### Experimental Site and Animal Management

A high-yielding Holstein dairy herd was enrolled in the study at the Shandong Yinxiang Weiye Group Company in Cao County, Shandong Provence, China (34°82′N, 115°54′E) for 20d. All study procedures were reviewed and approved by the Experimental Animal Welfare Ethical Committee, Institute of Animal Science, Chinese Academy of Agricultural Sciences (approval number IAS2020-99).

Three hundred and sixty nine cows were housed in a ventilated Free-Stall Barn. The Barn has 400 stalls, two pens (about 185 cows per pen). The cows have access to the outdoor, closed in the high-temperature summer, and they could see outside. The indoor stocking density was 14.52 m^2^ and 1.08 stalls per cow. The cowshed was equipped with fans and sprinklers for cooling. The cows were fed Total Mixed Rations (TMR) (08:30, 15:30, and 20:30) each after being milked (08:00, 15:00, and 20:00) three Times a day, *ad Libitum*. Each milking round lasted ~20 min. The TMR diet was formulated according to the lactation nutritional requirement of a 35 kg Milk/d Producing cow (NRC, 2001). Dietary ingredients and nutritional content are described in Table 1. The cow barn was equipped with an automatic manure scraper system, and recycled manure solids were used as bedding for dairy cows. The bedding was replaced, and the cow barn was disinfected thoroughly once a week to ensure hygienic cleanliness. The health of cows was checked by a veterinarian weekly.

**Table 1:** Ingredients and nutrient composition of experimental diets (% Air-dry basis).

**Item**	**Value**
**Ingredients**	**Content, %**
Alfalfa	10.39
Oat hay	2.42
Dandelion	0.48
Whole corn silage	48.33
Cottonseed	2.90
Beet pulp	2.42
Ground corn	7.49
Pressed corn	9.42
Soybean meal	8.70
Rapeseed meal	1.69
DDGS^a^	0.72
Extruded soybean	1.33
Mineral and vitamin mix ^b^	3.70
**Nutrient composition**	
CP	17.06
EE	3.32
NDF	35.75
ADF	18.20
NE_L_/(MJ/kg)	6.11

a *DDGS, Distillers Dried Grains with Solubles*.

b *Contained the following per kg of diets: VA 170 000 IU, VD 8 000 IU, VE 1 9000 IU, Ca 160 g, P 50 g, Fe 800 mg, Cu 680 mg, Mn 3 500 mg, Zn7 500 mg, Se 80 mg, I 400 mg, Co 38 mg*.

### Experimental Design and Treatments

A total of 359 second-parity Holstein lactating cows (days in milk = 136 ± 18, mean ± SD), housed in the same barn, were subjected to behavioral observations by two well-trained observers. Two observers conducted a three-day preliminary observation assessment on the farm simultaneously and on the same cow herd. The interobserver reliability points out an almost perfect agreement of the abnormal behavior assessment (PABAK ≥ 0.8) by calculation of prevalence-adjusted, bias-adjusted kappa (PABAK). Based on the results of the three-day preliminary observation, the 6 h (8:00–11:00, 14:00–17:00) with the highest incidence of tongue-rolling behavior in lactating cows were selected. During the experimental period, the dairy cows were observed for 7 days, 6 h a day, 3 times an hour, 10 min a time, a total of 126 times. Each round of scan sampling behavior observation (recording the activity of cows at pre-selected time intervals) was performed by two observers walking slowly from one end of the barn to the other with a walking speed of 3s/m to observe the cows (17). The numbers of cows corresponding to tongue-rolling behavior and other abnormal behaviors (Limp, Pica, Cross-sucking, Throwing, etc.) were recorded. The number of cows with tongue rolling behavior recorded in 126 observations was ranked. The top 10 cows with only tongue rolling behavior (all over 30 records) were selected as the tongue rolling group (TON) for further analysis, while 10 cows without any abnormal behavior in the same barn were randomly selected as the control (CON). After grouping, the cows in both groups were kept in the same original pen and marked with red or blue veterinary crayons.

Meteorological parameters (dry bulb temperature, T_db_, °C; and relative humidity, RH, %) in the barn were obtained from the Kestrel 5000 environmental meters (Nielsen-Kellerman Co., Boothwyn, USA) fixed to a barn post. The environmental meters were fixed at one-fourth, one-half and three-fourth of the barn, respectively, at a distance of 2 m from the ground. The environmental meters were measured at intervals of 5 min and THI were calculated using the formula of NRC (1971), as follows.


$$\begin{array}{l} \text{THI}=\left(1.8\times {\text{T}}_{\text{db}}\right)+32-\left(0.55-0.0055\times \text{RH}\right)\times \left(1.8\times \;{\text{T}}_{\text{db}}-26\right) \end{array}$$

The THI is divided into categories that potentially indicate the level of cow's heat stress according to Armstrong (18), which used THI <71 as a thermal comfort zone, 72 to 79 as mild heat stress, 80 to 90 as moderate heat stress, and >90 as severe heat stress.

### Sampling and Analysis

After cows were grouped and marked, 5 infrared supervision cameras (Hikvision Digital Technology Co., Ltd., Hangzhou, China) were used to record cow's behaviors for 24 h. The cameras were mounted to the opposite pen and ensured no blind spots in the field of view. The Observer XT software was used to analyze the behavioral data through the video. The time of standing, lying, feeding, and drinking of each marked cow was recorded using a continuous observation method.

Body condition score (BCS) was evaluated using a 5-point scale by two trained farmers with five year's experience, based on the research of Edmonson (19, 20).

Milk yield was recorded for consecutive days in a parallel milking parlor, and the daily mean was calculated for each cow. Milk samples were collected on the last third day at a volume ratio of 4:3:3 corresponding to the 08:00, 15:00, and 20:00 milking in 100 mL plastic vials. Samples were preserved with 2-bromo-2-nitropropan-1,3-diol and stored at 4°C. Milk protein, fat, and lactose were analyzed by the Dairy Quality Inspection Station of Beijing Dairy Cow Center (Beijing, China) with Master Pro 40SEC (Bulgaria). Milk protein yield and milk fat yield were calculated using the milk protein and fat content for the last third day multiplied by average milk yield during the study, respectively.

On a penultimate day, tail vein blood was collected into 6-mL heparin vacutainers (BD vacutainers, Fisher Scientific, Waltham, MA) at 06:00. Blood samples were centrifuged at 3,000 × g for 15 min at 4°C to isolate serum and stored at −80°C. Serum glucose (GLU), total protein (TP), albumin (ALB), alanine aminotransferase (ALT), aspartate aminotransferase (AST), lactate dehydrogenase (LDH), lactic acid (LD), triglycerides (TG), total cholesterol (TC), blood urea nitrogen (BUN) were measured using AU480 auto-analyzer (Olympus Co.). A BFM-96 multi-tube radioimmunoassay counter (Hefei, Anhui, CN) was used to determine the content of triiodothyronine (T3), thyroxine (T4), and cortisol (COR). The levels of dopamine (DA), serotonin (5-HT), epinephrine (E), norepinephrine (NE), γ-hydroxybutyric acid (GABA) and interleukin 6 (IL-6), interleukin 10 (IL-10), immunoglobulin A (IgA), immunoglobulin G (IgG), immunoglobulin M (IgM) in the samples were determined by biotin double antibody sandwich ELISA, following the manufacturer's instructions. All colorimetric data were measured using THERMO Multiskan Ascent (Waltham, MA, USA).

The ruminal fluid samples were collected using an oral stomach tube sampler 2 h after morning feeding on the last day. The first 100 ml collected fluid was discarded to prevent saliva interference. The following 50 mL ruminal fluid was filtered by four layers of gauze with a mesh size of 250 μm. The pH was measured immediately using a portable pH meter (PB-10, Sartorius, Germany). 20 mL of the samples were processed to analyze rumen volatile fatty acid (VFA) and Ammonia-N (NH3-N) after mixed with 0.4 mL of 50% sulfuric acid, stored at −80°C. The other part was stored in liquid nitrogen immediately. Individual and total VFAs were separated and quantified by gas chromatograph (GC-2010, Shimadzu, Kyoto). NH3-N concentration of the supernatant was measured by the indophenol method.

### DNA Extraction and 16s rDNA Gene Sequencing in Ruminal Fluid

Bacterial genomic DNA was extracted using a DNA extraction kit (B518225-0100) from rumen fluid. The concentration of DNA and the absorbance ratio at 260 nm (A260) and 280 nm (A280) were measured by NanoDrop2000 (NanoDrop Technologies, Wilmington, DE), and A260/A280 initially determined the DNA quality. The extracted DNA was run on 1% agarose gel electrophoresis to determine the DNA quality further. PCR amplified the V3-V4 variable region of the bacteria with the upstream primer sequence: 338F 5'- barcorde-ACTCCTRCGGGAGGCAGCAG-3', downstream primer sequence: 806R 5'- GGACTACHVGGGTWTCTAAT-3'. All samples underwent PCR with an initial denaturation step at 95°C for 3 min, followed by 30 s of denaturing at 95°C, 30 s of annealing at 50°C, and 45 s of extension at 72°C. This was repeated for 30 total PCR cycles and finished with a 10-min extension at 72°C.

The 16S rDNA gene sequencing procedure was performed through Illumina HiSeq 4000 platform (Illumina Inc., San Diego, CA, USA) following the manufacturer's instructions. The original sequences were deposited in the NCBI Sequence Read Archive (https://www.ncbi.nlm.nih.gov/sra/) under the accession number PRJNA777387.

The sequencing data analysis process was completed by QIIME2(https://docs.qiime2.org/2019.7/tutorials/overview/). The operational taxonomic units (OTUs, 97% similarity cutoff) were clustered using UPARSE (version 7.1, http://drive5.com/uparse/). The Greengenes database (http://greengenes.lbl.gov) is based on the RDP classifier algorithm annotated taxonomic information. Taxonomy was aligned by the RDP classifier against the SILVA (SSU115) 16S rDNA database (http://www.arb-silva.de/) using a confidence threshold of 70%.

Alpha diversity is applied in analyzing the complexity of species diversity for a sample through six indices, including, Chao1, Shannon, Simpson, ACE. All indices in our samples were calculated with QIIME (http://qiime.org/) software package and displayed with R software (Version 3.3.1, R Core Team, Vienna, Austria).

Beta diversity analysis was conducted to evaluate differences of samples in genus complexity. PCoA based on unweighted UniFrac distance metrics was conducted to compare bacterial profiles between the two groups. PCoA was measured using QIIME by stats package in R software. Microbiota diversity was calculated as the inverse Simpson diversity index based on the genus-level data.

### Statistical Analysis

Differences were assessed by students' *t* test. Results were presented as means ± SEM. *P* < 0.05 was defined as statistical significance. A normal distribution test of daily activity behavior, BCS, milk performance, ruminal fermentation parameters, and measured serum indicators data were first conducted using SAS procedure univariate with normal distribution option. The alpha diversity indices of bacterial communities and the relative abundances of bacterial taxa were compared between the TON and CON using the Student's *t*-test. Spearman correlation coefficients were calculated to assess the correlation between tongue rolling behavior and other significant indicators identified in this study using the NONPAR CORR procedure of SAS 9.4. Tongue rolling behavior was coded as a binary variable (0 = No; 1 = Yes) and BCS as a categorical variable, whereas the remaining data were continuous variables.

## Results

### Four Daily Activity Behaviors in 24h

**Figure 1:**
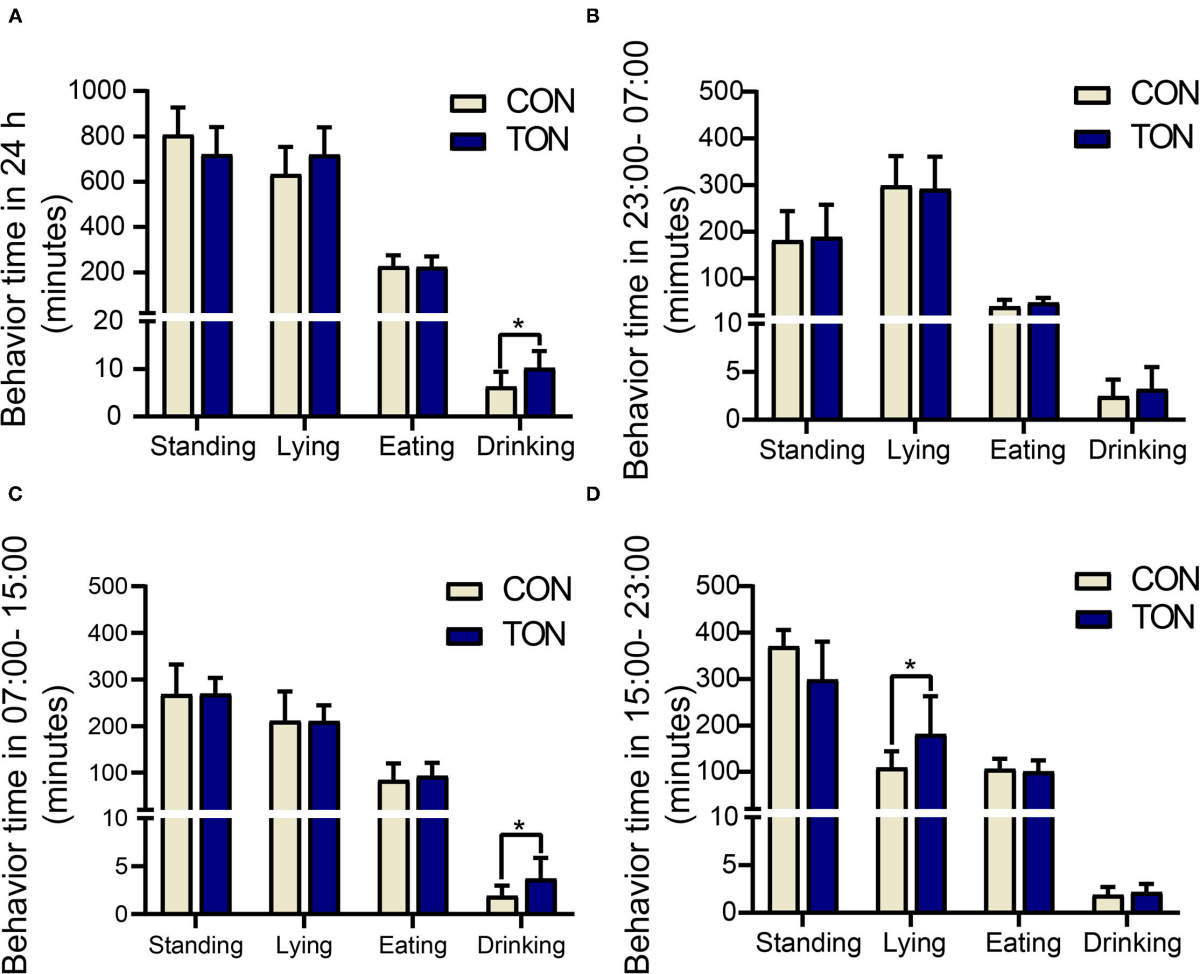
Minutes of activities in TON and CON. **(A)** Behavioral duration of TON and CON cows during 24 h. **(B)** Behavioral duration of TON and CON cows in 23:00-07:00. **(C)** Behavioral duration of TON and CON cows in 07:00-15:00. **(D)** Behavioral duration of TON and CON cows in 15:00-23:00. TON, cows with tongue rolling behavior; CON, cows without abnormal behavior. *P < 0.05.

Due to the fact that the cows in the pen were milked three times a day, the daily activity behavior time was recorded separately in 24h as three periods (8 h, milked to milked), as shown in Figure 1. The average temperature and humidity index (THI) was 68 in 23:00–07:00 (without heat stress response), 77 in 07:00–15:00 (low heat stress response), and 77 in 15:00–23:00 (low heat stress response).

Drinking time was counted when the cow's mouth touched the water and stopped when the mouth left the water. Drinking time (min/24 h) of the TON cows was significantly higher than the CON cows (*P* = 0.046). In the 7:00–15:00 period, the drinking time (min/8 h) in TON cows was significantly higher than that of CON cows (*P* = 0.034). And the lying time (min/8 h) in TON cows was significantly higher in the 15:00–23:00 period (*P* = 0.045).

**Figure 2:**
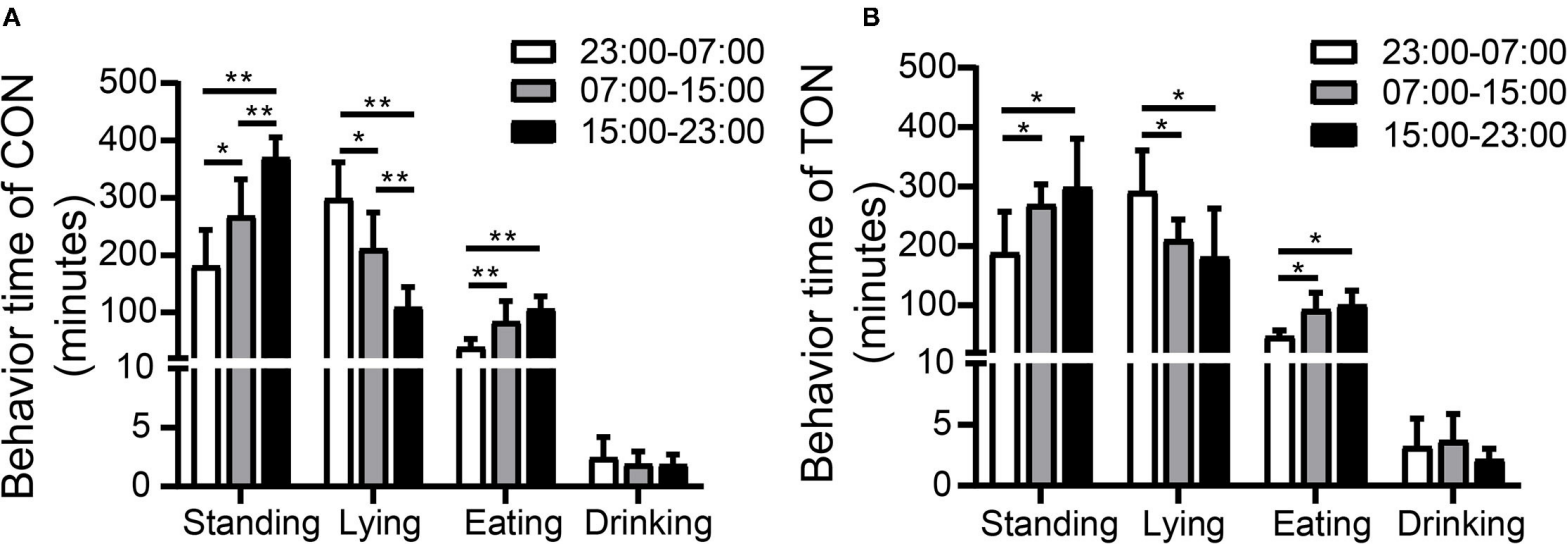
Minutes of activities duration among three periods in TON and CON. **(A)** Behavior duration of TON cows in three periods in 24 h. **(B)** Behavior duration of CON cows in three periods in 24 h. TON, cows with tongue rolling behavior; CON, cows without abnormal behavior. **P < 0.01; *P < 0.05.

Figure 2 showed the variations of the four behaviors comparing TON and CON cows during the three time periods, separately. In the TON cows, the lying time (min/8 h) seemed stable and was not different (*P* = 0.369) between the two periods with low heat stress (07:00–15:00 and 15:00–23:00). While the CON cows were more affected by heat stress and had significantly lower lying time (min/8 h) during the 15:00–23:00 time period (*P* = 0.002). The differences of lying and drinking time might indicate that TON cows have higher tolerance to the thermal environment.

### Production Performance and Body Condition Score

We then analyzed the production performance and BCS of the two groups, shown in Table 2. The daily yield of milk (*P* = 0.041), milk fat (*P* = 0.025), milk protein (*P* = 0.033), and lactose content (*P* = 0.002) were significantly higher in the TON group. Notably, the average daily milk yield, milk fat yield and milk protein yield of the TON group were 27.3, 41.2, 52.9% (9.953 kg) higher than that of the CON group, respectively. The average BCS of the TON cows was 2.70, which was significantly lower than that of CON cows (3.20, *P* <0.001).

**Table 2:** Milk yield, composition and body condition scores in dairy cow showing tongue- rolling behavior or no signs of abnormal behavior.

**Items**	**Experimental treatments^a^**		**SEM^b^**	***P*** **-value**
	**TON**	**CON**		
Milk yield^c^, kg/day	46.418	36.465	2.373	0.041
Milk fat, %	3.651	3.683	0.175	0.932
Fat yield, kg/day	1.768	1.252	0.119	0.025
Milk protein, %	3.141	3.221	0.045	0.392
Protein yield, kg/day	1.794	1.173	0.078	0.033
Lactose, %	5.248	5.078	0.028	0.002
BCS^d^, 1 to 5 scores	2.700	3.200	0.068	<0.001

a *TON, tongue-rolling cows; CON, normal cows, no abnormal oral behavior*.

b *SEM, standard error of the mean*.

c *Milk yield, average milk yield of study*.

d *BCS, body condition scores, 1–5 scores*.

### Rumen Fermentation Parameters

Ruminal fluid pH (*P* = 0.044) and total VFAs (*P* = 0.035) of the TON cows were significantly lower. The ratio of acetate to propionate in TON cows was significantly higher than the CON cows (*P* = 0.028), and the propionate (*P* = 0.001) and valerate (*P* = 0.015) content were significantly lower (Table 3).

**Table 3:** Ruminal fermentation parameters in dairy cow showing tongue- rolling behavior or no signs of abnormal behavior.

**Items**	**Experimental treatments^a^**		**SEM^b^**	***P*** **-value**
	**TON**	**CON**		
pH	6.246	6.419	0.043	0.044
Ammonia-N (mg/dL)	18.366	19.334	0.773	0.556
Acetate (mmol/L)	68.731	71.972	4.458	0.281
Propionate (mmol/L)	27.272	32.025	0.812	0.001
A/P^c^	2.520	2.247	0.159	0.028
Butyrate (mmol/L)	12.572	13.675	0.410	0.203
Isobutyrate (mmol/L)	0.703	0.7875	0.030	0.179
Valerate (mmol/L)	1.575	1.866	0.061	0.015
Isovalerate (mmol/L)	1.125	1.241	0.058	0.349
TVFA (mmol/L)	111.978	121.566	2.267	0.035

a *TON, tongue-rolling cows; CON, normal cows, no abnormal oral behavior*.

b *SEM, standard error of the mean*.

c *A/P, acetate/propionate*.

### Ruminal Bacteria Diversity

**Figure 3:**
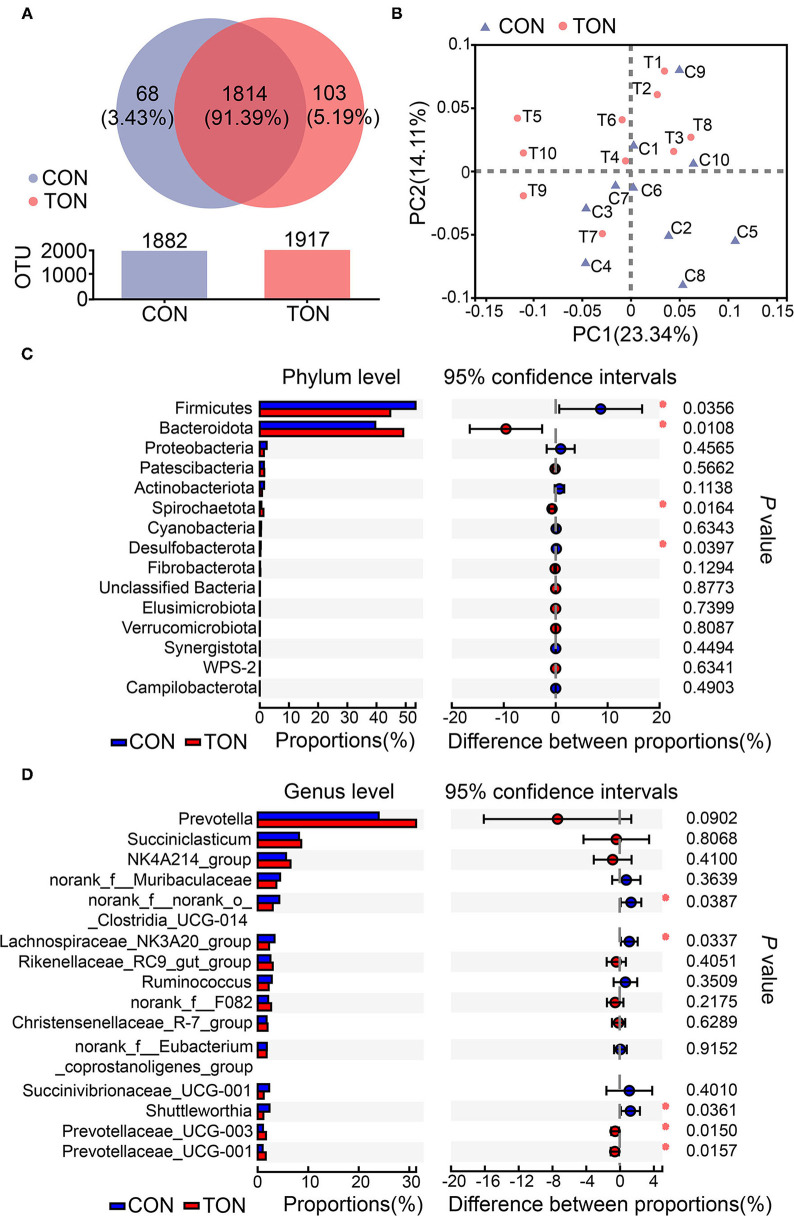
**(A)** The Venn diagram of unique or shared OTUs in the TON and CON groups. Different colors represent different groups, the number in the overlapping part represents the number of species shared by multiple groups, and the number in the non-overlapping part represents the number of species unique to the corresponding group. **(B)** Principal component analysis (PCoA) of ruminal bacteria community in TON and CON groups. PC1, the first principal component; PC2, the second principal component. **(C)** Student's *t-*test bar plot on Phylum level. **(D)** Student's *t*-test bar plot on Genus level. TON, cows with tongue rolling behavior; CON, cows without abnormal behavior.

According to previous studies, low roughage intake may lead to tongue-rolling behavior. Cows with tongue-rolling behavior may have altered rumen microflora due to insufficient roughage intake. We further analyzed the causes of ruminal fermentation changes at the aspect of rumen bacterial diversity. The sequencing reads number of all samples was between 30,000 and 45,000, and the mean length of all reads was more than 430 nt. Totally, 1,985 OTUs, 19 phyla, and more than 259 genera were identified in the present study, and the unique or shared OTUs in the TON and CON groups were shown in the Venn diagram (Figure 3A). All the taxonomy information is displayed in Supplementary Material 1.

Alpha-diversity is applied in analyzing the complexity of species diversity for a sample through OTUs, Chao1, Shannon, Simpson, Ace, Coverage. All these indices of α-diversity were shown in Table 4 but not significant between TON and CON groups.

**Table 4:** Number of observed species, richness and diversity indices in rumen microbes of dairy cow showing tongue- rolling behavior or no signs of abnormal behavior.

**Items**	**Experimental treatments^a^**		**SEM^b^**	***P*** **-Value**
	**TON**	**CON**		
OTUs^c^	1195.4	1149.5	25.388	0.380
Shannon	5.588	5.503	0.057	0.475
Simpson	0.013	0.019	0.004	0.416
ACE^d^	1428.665	1383.2	28.380	0.438
Chao1	1438.626	1397.057	30.079	0.504
Good's Coverage	0.988	0.988	0.0003	0.796

a *TON, tongue-rolling cows; CON, normal cows, no abnormal oral behavior*.

b *SEM, standard error of the mean*.

c *OTUs, operational taxonomic units*.

d *ACE, abundance-based coverage estimator*.

As shown in Figure 3B, PCoA axes 1 and 2 accounted for 23.34 and 14.11% of the total variation, respectively. Based on the results, the bacteria community in the TON cows could be separated from the CON cows by PCoA except C9.

Differential analysis on ruminal bacteria at different levels was then conducted to investigate the effect of bacterial abundance on rumen fermentation. The results in the level of phylum and genus (the species with the top 15 abundances) are shown in Figures 3C, D, respectively. *Firmicutes* and *Bacteroidetes* contribute to the two most abundant phylum of ruminal bacteria. On top 15 phylum, the abundances of *Firmicutes* and *Desulfobacterota* in the TON group were significantly lower (*P* = 0.036, *P* = 0.040, respectively). The abundance of *Bacteroidetes* and Spirochaetota were significantly higher than that in the CON group (*P* = 0.010, *P* = 0.016, respectively). At the genus level, *Prevotella, Succiniclasticum, Ruminococcaceae NK4A214_group* were the first three abundant genus. In particular, there was a tendency for *Prevotella* to increase significantly in the TON group (*P* = 0.090). These results indicated that the proportion of rumen bacteria used to decompose roughage decreased in TON cows.

### Serum Biochemistry and Inflammatory Indicators

The serum stress hormone indicators of the cows in the TON cows, including T3 (*P* = 0.007), T4 (*P* = 0.001), NE (*P* = 0.019), COR (*P* = 0.001), and 5-HT (*P* = 0.042), were significantly higher than those of CON cows, and serum adrenaline tended to increase (*P* = 0.062; Table 5). The LDH (*P* = 0.019) and serum glucose (*P* = 0.044) levels of the TON cows increased significantly, accompanied by a higher TG (*P* = 0.016) and TC (*P* = 0.023) concentration. Similarly, the contents of serum total protein (*P* = 0.019), albumin (*P* < 0.001), and IL-6 (*P* = 0.048) of the TON cows were significantly higher than those of CON cows. Serum LD (*P* = 0.007) concentration in TON cows was significantly lower than in the CON cows. These results indicated that TON cows had an increased degree of stress, metabolic status, and inflammation.

**Table 5:** Comparison of differences in blood physiological and biochemical indices in dairy cows showing tongue- rolling behavior or no signs of abnormal behavior.

**Items^c^**	**Experimental treatments^a^**	**SEM^b^**	***P*** **-value**
	**TON**	**CON**		
Glucose, mmol/L	3.340	3.103	0.058	0.044
LDH, U/L	225.778	168.400	12.207	0.019
LD, mmol/L	0.975	1.343	0.810	0.007
TG, mmol/L	0.170	0.136	0.007	0.016
TC, mmol/L	7.063	5.888	0.261	0.023
Urea nitrogen, mmol/L	3.144	3.093	0.135	0.861
T3, ng/mL	3.229	2.571	0.126	0.007
T4, ng/mL	329.316	171.139	26.200	0.001
COR, ng/mL	13.943	8.835	0.864	0.001
DA, nmol/L	20.710	21.579	0.588	0.492
5-HT, pg/mL	542.409	480.434	15.044	0.042
E, ng/mL	3.432	3.190	0.064	0.062
NE, ng/mL	19.086	16.371	0.587	0.019
GABA, umol/L	1.229	1.259	0.029	0.638
Total Protein, g/L	67.920	64.110	0.814	0.019
Albumin, g/L	43.360	37.540	0.803	<0.001
AST, U/L	93.556	87.333	2.555	0.248
ALT, U/L	38.200	36.300	0.969	0.353
IL-6, ng/L	422.612	377.291	11.311	0.048
IL-10, pg/mL	22.202	21.406	0.738	0.612
IgA, ug/ml	114.160	110.793	3.186	0.620
IgG, mg/ml	3.738	3.666	0.121	0.781
IgM, mg/ml	0.981	0.990	0.031	0.894

a *TON, tongue-rolling cows; CON, normal cows, no abnormal oral behavior*.

b *SEM, standard error of the mean*.

c *GLU, serum glucose; TP, total protein; ALB, albumin; ALT, alanine aminotransferase*.

*AST, aspartate aminotransferase; LDH, lactate dehydrogenase; LD, lactic acid; TG, triglycerides; TC, total cholesterol; BUN, blood urea nitrogen; T3, thyroid hormones 3; T4, thyroid hormones 4; COR, cortisol; DA, dopamine; 5-HT, serotonin; E, epinephrine; NE, norepinephrine; GABA, γ-hydroxybutyric acid; IL-6, interleukin 6; IL-10, interleukin 10; IgA, immunoglobulin A; IgG, immunoglobulin G; IgM, immunoglobulin M*.

### Spearman Correlation of Tongue Rolling Behavior

**Figure 4:**
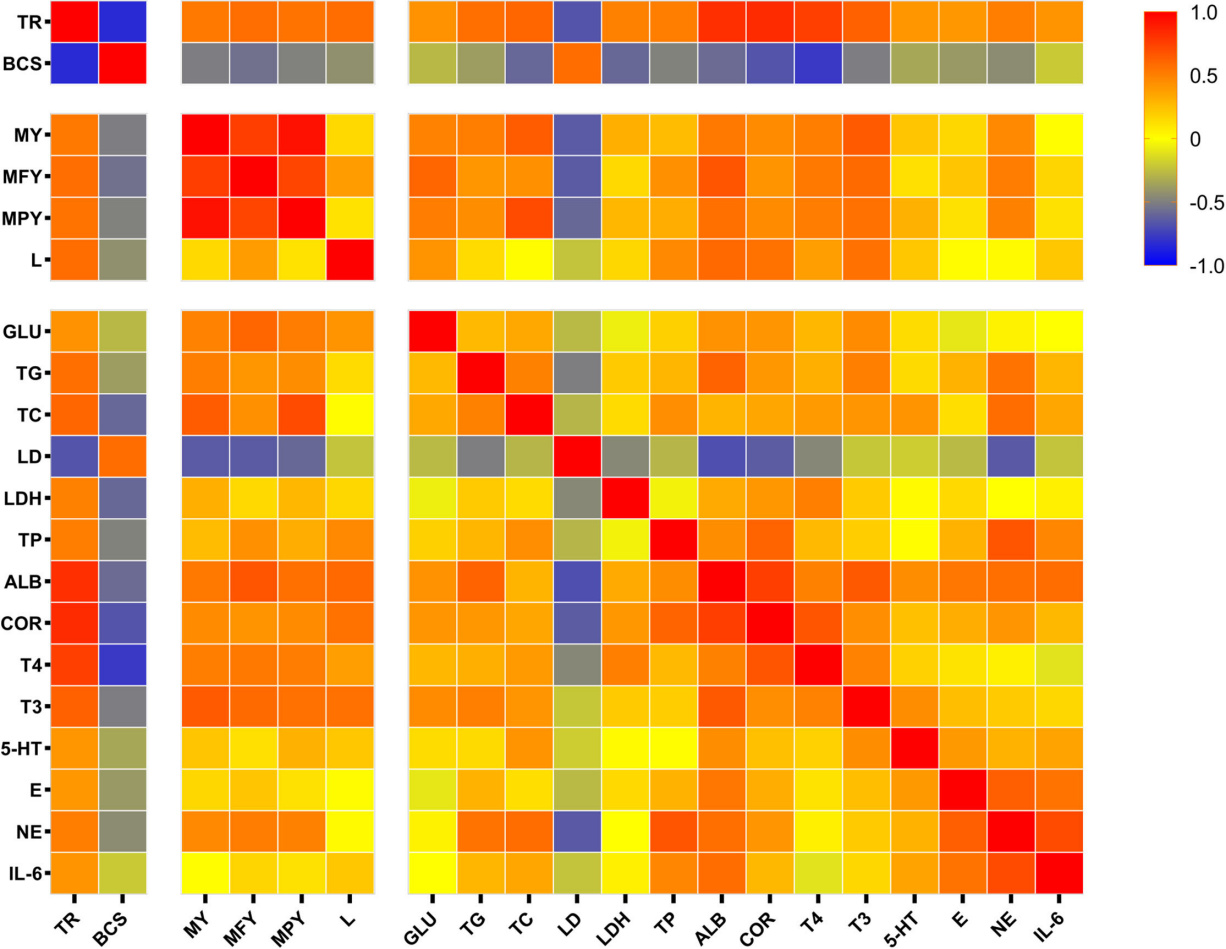
Spearman correlation matrix with r values, from log2 fold change values of the expression of tongue rolling behavior, body condition score, production performance and selected serum indicators in dairy cows. Red indicates a strong positive correlation, blue indicates a strong negative correlation and yellow indicates no correlation. TR, tongue rolling; BCS, body condition score; MY, milk yield; MFY, milk fat yield; MPY, milk protein yield; L, lactose; GLU, Serum glucose; TG, triglycerides; TC, total cholesterol; LD, lactic acid; LDH, lactate dehydrogenase; TP, total protein; ALB, albumin; COR, cortisol; T4, thyroxine; T3, triiodothyronine; 5-HT, serotonin; E, epinephrine; NE, norepinephrine; IL-6, interleukin 6.

In the present study, a Spearman correlation heatmap for partial significant indicators detected was generated to examine the correlations between tongue rolling and milk performance, serum ingredient consents for metabolism and stress (Figure 4). The TON cows exerted remarkable changes on body condition and production performance. Tongue rolling behavior was positively correlated with milk yield (*r* = 0.525, *P* = 0.025), lactose content (*r* = 0.572, *P* = 0.001) and had a strongly negative correlation with BCS (*r* = −0.839, *P* < 0.0001) and serum LD (*r* = −0.674, *P* = 0.003). Serum stress and immune indicators including ALB (*r* = 0.815, *P* < 0.0001), COR (*r* = 0.830, *P* < 0.0001), T3 (*r* = 0.616, *P* = 0.005), T4 (*r* = 0.751, *P* < 0.0001), NE (*r* = 0.505, *P* = 0.039), E (*r* = 0.404, *P* = 0.086) and TP (*r* = 0.503, *P* = 0.024) were positively correlated with tongue rolling behavior. Tongue rolling behavior was moderately positively correlated with serum glucose (*r* = 0.425, *P* = 0.062), TG (*r* = 0.561, *P* = 0.019), TC (*r* = 0.597, *P* = 0.082), LDH (*r* = 0.490, *P* = 0.033).

## Discussion

Tongue rolling behavior is often considered a sign of psychological or behavioral frustration in cows (21). Cows exhibiting tongue rolling behavior seem to be caused by inhibition of critical oral activities such as sucking, grass foraging, and chewing (22). In general, tongue-rolling behavior was only analyzed as qualitative data, characterized as either present or absent, with no specific and clear explanation of why the behavior arises (23) and what alters in the cows. In this experiment, we observed that the lactating cows with high-frequency tongue rolling behavior have significant differences in the time of lying and drinking behaviors, milk producing, BCS, and metabolic stress levels.

### Changes of Ruminal Fermentation and Bacteria Diversity

The content and composition of ruminal VFA were affected by dietary type or concentrate-to-roughage ratio (mainly) (24), feed processing (25), microbial species and combinations (26, 27), rumen pH (28) and feeding pattern (times) (29). The decrease of rumen pH would also affect the composition of microorganism and reduce the output and proportion of VFAs. In this study, the two groups of cows had the same feed and management background. The pH of the ruminal fluid in the TON group and the CON group were within the normal range (pH 5.5~6.8) (30). Russell (31) found that cows fed 90% concentrate had lower ruminal pH values (6.22 vs. 6.86), higher VFAs concentrations (85 vs. 68 mM), and lower acetate to propionate ratios (2.24 vs. 4.12) than cows fed forage only. Lechartier et al. (32) found that a lower daily roughage level would reduce the average pH of stomach fluid and the acetate to propionate ratio. Nevertheless, we found that the TON group had lower pH and total VFAs concentration of the ruminal fluid than the CON group when fed the same TMR. Moreover, the acetate to propionate ratio of the TON group increased, which was not consistent with the mentioned reports. It suggested that the variation in pH and VFAs in the TON group was not due to the feed processing but for other reasons in the rumen. The appropriate rumen environment was mainly attributed to the stabilized ruminal microbial community (33). Bacteria from the *Bacteroidetes* and *Firmicutes* phyla dominated the core microbiome in both groups, consistent with the report of Golder (34), but the proportions have changed significantly. At the phylum level, the TON cows had a lower abundance of *Bacteroides* and *Firmicutes* than the CON cows. At the genus level, the abundance of *Prevotella* in the TON cows was also significantly higher than the CON cows. It indicated the TON cows had a lower proportion of the cellulolytic bacteria, which may decline the fiber degradation in the rumen, thus leading to inadequate decompose of roughage. Since the higher ratio of polysaccharide and protein degrading bacteria, the ability to decompose concentrated feed of TON cows was higher, consistent with Fernando's studies feeding various forages and concentrates at different ratios (35). Based on the rumen indicators above, we inferred that the ruminal digestibility of the roughage of TON cows was weaker than that of the CON cows. Saliva is an important functional substance for rumen fermentation. Castillo-Lopez et al. (36) pointed out that salivary secretions are essential for regulating digestive processes. Ridge et al. (37) mentioned that the ruminating time of grazing dairy cows is as high as 10.2 h/day, while captive dairy cows spent 3.8–9.4 h/day ruminating. When cows digest high-concentration feed, the rumen environment will undergo rapid physiological and chemical changes. The dairy cows may then deal with this discomfort through more oral behaviors to produce saliva and increase rumen buffer utilization (38). Potential picky feeding behavior on roughage might lead to inadequate roughage intake, which in turn leads to inadequate ruminant and salivary secretion in cows. An insufficient feeding of roughage would result in less ruminant time and less saliva entering the rumen. Overall, the occurrence of tongue-rolling behavior might be related to ruminating behavior and saliva secretion. In this study, ruminal fermentation and bacteria diversity were different comparing TON cows and CON cows. However, we tend to believe that tongue-rolling behavior is an external manifestation of a specific type of cows rather than a cause of the differences between the two groups.

### Changes of Milk Performance, Metabolism and Body Condition Score

In the experiment the cow's tongue rolling behavior is positively correlated with milk production, which was consistent with Dantzer's research (39). Milk production performance and BCS are closely related to economic benefits. Animal nutrition has a majority impact on milk composition (40). When the nutritional needs of dairy cows are higher than the actual intake of nutrients, the number of nutrients required will significantly exceed the amount of feed consumed. At this time, fat and protein stored in the body will be used to meet milk production needs, and dairy cows will have a negative balance of protein and fat (41). From the appearance of view, a negative balance of nutrients can lead to emaciation in cows over time. Cow's BCS was associated with risks to animal health (42). The TON cows with high milk performance were accompanied by lower BCS, which might be consistent with the negative balance of nutrients. We further searched the differences from the perspective of serum physiologic and metabolic indicators. Cows with high production must meet a tremendous metabolic challenge to provide enough glucose to support milk production. Changes in blood parameters were mainly caused by changes in energy and protein metabolism, and were related to milk production levels (43). The level of T4 was positively associated with plasma glucose concentration, lactose, and milk output, which might increase metabolism of glucose via glycolysis and involve increased mammary utilization of pre-formed long-chain fatty acid (44). The increasing content of serum T3, T4 indicated that the TON cows had higher metabolism levels (45, 46). Serum glucose is one of the dietary factors that affect milk synthesis (47), mainly derived from gluconeogenesis and glycogen catabolism (48, 49). Lactose, as the main component that regulates the osmotic pressure of milk, is the main factor determining milk production (50). And glucose is the primary precursor of milk lactose, which can induce the growth of milk cow mammary epithelial cells and lactose synthesis. The correlation between milk yield, glucose levels, and milk lactose concentration in cows exhibiting tongue-rolling behavior was consistent with previous reports of correlated indicators in high-yielding cows.

The liver coped with an increasing supply of TC and TG by enhancing oxidation or reesterification (51). Milk production and serum glucose are important for serum total cholesterol levels (52). The present study supports these results because a positive correlation between serum TC levels and milk yield was observed, as well as a positive association for TC with glucose. In addition, the tongue rolling behavior in the study was positively linked with measurements of glucose, TG, TC, T3, and T4 in serum, showing the potential role of these metabolites in affecting the behavioral expression of cows.

On the one hand, due to the relative shortage of nutrition supplements or digestion in rumen of TON cows, the quantity of rumen available nutrient could not meet the needs of the metabolic alterations of high energy demand and lactation synthesis, which might increase the degradation of protein and fat reserves and lead to a risk of negative body balance (41).

Nevertheless, the mentioned serum nutrition and metabolism indicators were all within the normal expected limits for dairy cows fed. Therefore, this suggests that the lower BSC of TON cows might be due to the mobilization of their glycogen, protein, and other nutrients, reducing their nutrient accumulation to produce more milk.

### Posture and Activity Behaviors

Since there was no significant change in the feeding time of the two groups within 24 h, we speculated that the tongue rolling behavior is unlikely to be caused by oral diseases. Dairy cows have a strong desire to lie down after feeding. Adequate lying time generally indicates that the cow is in a good welfare situation (53). During heat stress, behavioral responses have been observed in cows such as increased standing time and shade seeking, reduced activity and movement, and changes in water and feed intake patterns (54). When the THI was above the comfort threshold (≥72), cows would spend less time lying and ingesting (55). Fregonesi and Leaver (56) reported that high-yield dairy cows had a shorter lying time than low-yield cows. In the study, we observed that the lying time of the CON cows gradually decreased with THI increasing. At the same time, the TON cows have stable lying time and increased drinking time at the same duration, which was inconsistent with the previous report. Combining the results above, we speculate that more drinking and lying time in TON cows might relate to their stronger ability of heat tolerance.

### Stress and Inflammation

Endogenous opioid peptides participate in the formation, transmission, modulation, and perception of pain signals (57). When animals encounter difficult conditions, endogenous opioids are often released (58). There is evidence that endogenous opioids play a key regulatory role in basal ganglia direct and indirect pathways, and increased inhibition (*via* mu-opioid receptors) of the indirect (dorsal striatopallidal) pathways might increase the occurrence of spontaneous stereotypical behavior in animals (59). And stereotypy could increase levels of central endogenous opioid thereby allowing the animal to 'cope' with stressful environments and events (60). Tongue-rolling behavior may transfer environmental stress and increase the ability of cows to adapt to the captive environment, consistent with the results of Briefer's research on horse stereotypes to reduce stress (61). Prodanovic et al. (62) found that tongue rolling behavior was only among cows with a deviation of biochemical parameters values, which pointed out a possible connection between the tongue rolling behavior and biochemical composition of blood. The serum concentration of COR, 5-HT, E, and NE are generally considered biomarkers of cow stress, and they are positively correlated with the level of animal stress. In this study, we found that the behavior of tongue rolling is closely related to the stress level and metabolic condition of cows. It illustrated that tongue rolling might be an external behavioral manifestation of high-stressed metabolism in dairy cows.

Long-term stress in dairy cows often causes inflammation, accompanied by abnormal behaviors. Li et al. (63) found that high THI stress in summer would increase levels of inflammatory indicators and induce different degrees of oxidative stress, inflammation response, and stress hormone imbalances on lactating dairy cows. Trevisi et al. (64) found that stress could activate inflammatory indexes of dairy cows. An increase in stress hormones leads to a shift in cytokine homeostasis toward a Th2 profile (65), resulting in increased production of Th2 cytokines, including IL-6, which is consistent with our results of the TON cows. As the results described, the higher inflammatory indicators of the TON cows indicated an increase in proinflammatory signaling within blood and liver (66).

### Pathophysiology Perspectives of Stereotypies

The hedonic sensation elicited by natural reward substrates and behaviors might be the source of stereotypical behavior (67). Dopamine and dopaminergic pathways have been identified as the primary underlying substrates of stereotypy development and maintenance (68, 69), and endogenous opioids may have a substantial role to play in the causal and functional aspects of this behavioral condition. Chronic stimulation with natural reward substrates upregulates opioid receptors within the striatum, increasing the incidence of stereotypic behavior (70). Researchers found that opioid antagonists could transiently eliminate stereotypic behavior (71, 72). For stereotypy cows, however, the drug residues of opioid antagonists may affect the quality and safety of dairy products, which is debatable. In addition, Galizzi Vecchiotti and Galanti (73) proposed that equine stereotyped behavior is hereditary that these horses have inherited a tendency to perform a particular stereotypy. Hemmann et al. (74) studied case–control cohorts of horses with crib-biting behavior in two breeds to test the possible association of eight candidate genes (Leptin, Ghrelin, Ghrelin receptor, Dopamine receptor, Serotonin receptor, μ-opioid receptor, N-cadherin and Semaphorin, which were associated with the occurrence of certain stereotypic behaviors in animals such as mice, pigs, and humans). But there was no reveal evidence for an association at any of the tested loci in the target breeds. Therefore, different types of spontaneous stereotypic behavior may have different neurological foundations within or across species (59). The neural basis of tongue-rolling behavior in cows also needs further targeted research.

## Conclusions

Though our behavior observational study, it seems that cows with high-frequency tongue rolling behavior have higher production performance and activated metabolic status accompanied by higher stress. Therefore, tongue-rolling behavior might be an additional behavior indicator for farmers to assume the physical condition of dairy cows.

## Data Availability Statement

The datasets presented in this study can be found in online repositories. The names of the repository/repositories and accession number(s) can be found in the article/Supplementary Material.

## Ethics Statement

The animal study was reviewed and approved by the Experimental Animal Welfare Ethical Committee, Institute of Animal Science, Chinese Academy of Agricultural Sciences. Written informed consent was obtained from the owners for the participation of their animals in this study.

## Author Contributions

FS, XG, and GZ designed the study. FS, QZ, and XC conducted the experiment. FS performed lab analysis and wrote the manuscript. FS, XG, and XC performed statistics and analyzed the data. XG and XC revised the article. All authors carefully read and approved the final revision of the manuscript.

## Funding

The study was financially supported by the Beijing Dairy Industry Innovation Team Project (BAIC06-2021; Beijing, China) and the Agricultural Science and Technology Innovation Program (ASTIP-IAS07; Beijing, China). The funders had no role in the study design, the data collection and analysis, the decision to publish, or the preparation of the manuscript.

## Conflict of Interest

The authors declare that the research was conducted in the absence of any commercial or financial relationships that could be construed as a potential conflict of interest.

## Publisher's Note

All claims expressed in this article are solely those of the authors and do not necessarily represent those of their affiliated organizations, or those of the publisher, the editors and the reviewers. Any product that may be evaluated in this article, or claim that may be made by its manufacturer, is not guaranteed or endorsed by the publisher.
